# Selective and low temperature transition metal intercalation in layered tellurides

**DOI:** 10.1038/ncomms13809

**Published:** 2016-12-14

**Authors:** Takeshi Yajima, Masaki Koshiko, Yaoqing Zhang, Tamio Oguchi, Wen Yu, Daichi Kato, Yoji Kobayashi, Yuki Orikasa, Takafumi Yamamoto, Yoshiharu Uchimoto, Mark A. Green, Hiroshi Kageyama

**Affiliations:** 1 Department of Energy and Hydrocarbon Chemistry, Graduate School of Engineering, Kyoto University, Nishikyo-ku, Kyoto 615-8510, Japan; 2 Institute of Scientific and Industrial Research, Osaka University, Ibaraki, Osaka 567-0047, Japan; 3 CREST, Japan Science and Technology Agency (JST), Chiyoda-ku, Tokyo 102-0076, Japan; 4 Graduate School of Human and Environmental Studies, Kyoto University, Sakyo-ku, Kyoto 606-8501, Japan; 5 School of Physical Sciences, University of Kent, Canterbury, Kent CT2 7NH, UK

## Abstract

Layered materials embrace rich intercalation reactions to accommodate high concentrations of foreign species within their structures, and find many applications spanning from energy storage, ion exchange to secondary batteries. Light alkali metals are generally most easily intercalated due to their light mass, high charge/volume ratio and in many cases strong reducing properties. An evolving area of materials chemistry, however, is to capture metals selectively, which is of technological and environmental significance but rather unexplored. Here we show that the layered telluride *T*_2_PTe_2_ (*T*=Ti, Zr) displays exclusive insertion of transition metals (for example, Cd, Zn) as opposed to alkali cations, with tetrahedral coordination preference to tellurium. Interestingly, the intercalation reactions proceed in solid state and at surprisingly low temperatures (for example, 80 °C for cadmium in Ti_2_PTe_2_). The current method of controlling selectivity provides opportunities in the search for new materials for various applications that used to be possible only in a liquid.

## Abstract (web summary)

The intercalation of metal ions in layered structures has a wide variety of applications, from energy storage to environmental remediation. Here the authors report that layered telluride structures can selectively bind transition metals and this intercalation can occur in solid state at mild conditions.

Intercalation compounds allow incorporation or exchange of foreign atoms or molecules into the voids of various topologies in the host lattices such as cages (zeolites and so on)^1^, channels (h-WO_3_ and so on)^2^ and two-dimensional (2D) spaces (graphite and so on)^3^ and represent an important frontier in solid state chemistry. The structural diversity of these solids gives rise to a vast array of applications too extensive to summaries. However, two key areas of particular note are those materials serving as reservoirs to store and release alkali metal ions, for example, Li^+^ for high performance for energy storage devices^4^ and the tuning of exotic superconductivity in Na_*x*_CoO_2_·1.3H_2_O, Li_*x*_(THF)_*y*_HfNCl (THF, tetrahydrofuran) and Cu_*x*_Bi_2_Se_3_ upon intercalation^5^,^6^,^7^.

The rich intercalation chemistry that has been discovered for layered materials with van der Waals (vdW) interactions, which includes V_2_O_5_, *M*NCl (*M*=Ti, Zr), *MX*_2_ (*M*=Ti, Zr, Ta and so on; *X*=S, Se), *M*P*X*_3_ (*M*=Mg, Fe, Ni and so on; *X*=S, Se), MoS_2_ and *M*O*X* (*M*=Ti, V, Fe and so on; *X*=Cl, Br)^8^,^9^,^10^,^11^,^12^, yields various chemical and physical properties. Rather weak interlayer (vdW) interactions and a flexible interlayer spacing allows for incorporation of not only the lowest charged small alkali metal cations, but also many other heavier metals in the periodic table. For instance, TaS_2_ is capable of intercalating alkali metals, alkali earth metals and nearly all 3*d* transition metals as well as organic amines^13^,^14^,^15^,^16^,^17^,^18^,^19^. However, the poor selectivity in these materials hinders the preferential sorption of heavy metals, which is of environmental significance in the remediation of important toxic heavy metals pollutants, such as Cd, Pb and Hg^20^. Traditional absorbents and ion-exchangers like activated carbon, clays and zeolites also suffer from this problem^21^,^22^,^23^.

Recently, Kanatzidis and colleagues have demonstrated that several layered sulfides exhibit highly selective ion-exchange properties for Sr, Hg, Pd and Cd within K_2*x*_Mn_*x*_Sn_3–*x*_S_6_ (0.5<*x*<0.95; refs 24, 25) and Cs within [(CH_3_)_2_NH_2_]_2_Ga_2_Sb_2_S_7_·H_2_O (ref. 26), via an aqueous solutions with excess lighter alkali metal ions and protons. In both systems, the high selectivity is ascribed to the favourable interactions between the soft Lewis base S^2–^ ions of the host layer and the soft Lewis acid of the metal ions as a guest. In this study, we utilize the smaller electronegativity of tellurium to provide a softer Lewis base, compared with O, S and Se^27^, within the layered tellurides, Ti_2_PTe_2_ and Zr_2_PTe_2_, which we expect will promote a higher degree of covalency to selectively bind heavy metals. Prior intercalation studies are largely confined to binary tellurides such as TiTe_2_, ZrTe_2_ and IrTe_2_, with a primary interest in superconductivity and ferromagnetism^28^,^29^. The structure of *T*_2_PTe_2_ (*T*=Ti, Zr) is built up of slabs of hexagonal closed-packed triple Te–P–Te layers with *T* atom being octahedrally coordinated by three Te and three P atoms (see Fig. 1a)^30^,^31^. The individual slabs are stacked such that a rhombohedral structure is formed. It is isostructural with 3R-type Ta_2_CS_2_ (ref. 32). The absence of metal species between the double Te layers suggests relatively weak interlayer interactions and thus intercalation chemistry.

Herein, we show that *T*_2_PTe_2_ displays exclusive insertion of transition metals of Cu, Zn and Cd with tetrahedral coordination preference to tellurium. The intercalation reactions proceed in solid state and at surprisingly low temperatures—as low as 80 °C for cadmium in Ti_2_PTe_2_ and 70 °C for copper in Zr_2_PTe_2_. The metal intercalation induces a structural transition involving the change in stacking sequence from the 3R- to the 1T-type. Our first-principles calculations demonstrate a unique band structure with a pseudogap just above the Fermi energy, implying that the selective intercalation originates predominantly from the thermodynamic stabilization of the intercalated phase. The observed low temperature solution-free metal capturing, together with the reversible reaction enabling separation of hazardous metal from a powder metal mixture or an alloy, suggest potential applicability of our materials in environmental remediation.

## Results

### Metal intercalation and structural characterization

We tested a series of alkali, alkali earth, transition and post-transition metals (*M*) for their intercalation properties into Ti_2_PTe_2_. The reactions were typically conducted by heating a pelletized mixture of Ti_2_PTe_2_ and *M* in vacuum at temperatures up to 400 °C (see the ‘Methods' section for a detailed synthesis procedure). Powder X-ray diffraction patterns collected on the samples after attempted intercalation revealed little or no intercalation in most cases, including alkali metals, as summarized in Supplementary Fig. 1. These reactions either lead to decomposition of Ti_2_PTe_2_ or no reaction was observed. In contrast, Zn, Cu and Cd readily intercalated. Fe and Mn intercalation occurred only at high temperature (400 °C), yet leaving a large amount of Ti_2_PTe_2_ unreacted (Supplementary Fig. 2). Zr_2_PTe_2_ shows the same reaction result, except the fact that no intercalation was observed for Fe and Mn (Supplementary Fig. 1).

As a representative example, we first show the intercalation of Zn into Ti_2_PTe_2_. The product prepared at 300 °C for 48 h with a Zn/Ti_2_PTe_2_ molar ratio (*p*) of 0.4 exhibits a similar X-ray diffraction profile to the parent phase (Fig. 2). However, a close inspection of the pattern shows a significant shift of the (00*l*) reflections. Moreover, a clear change in stacking sequence from the rhombohedral to a primitive cell is evident from the appearance of new characteristic reflections. Here, the lattice constants for the precursor (R) and product (P) phases are correlated by the relation: *a*_P_≈*a*_R_, 3*c*_P_≈*c*_R_. The clear expansion of the normalized *c* axis (*c*_P_–*c*_R_/3=0.348 Å) suggests successful intercalation of Zn, presumably between the double Te layers. As shown in Supplementary Fig. 3, energy dispersive X-ray spectroscopy (EDX) measurements show that the Zn content is approximately *x*≈0.36(5) (in Zn_*x*_Ti_2_PTe_2_). The identical Bravais lattice change from R to P along with the elongated normalized *c* parameter is also observed for Cd (Fig. 2), Cu, Fe and Mn in Ti_2_PTe_2_ (Supplementary Fig. 2), and Cd, Zn and Cu in Zr_2_PTe_2_ (Supplementary Fig. 4). We note that Fe and Mn intercalated phases for *T*=Ti were not obtained as a single phase, but together with a large amount of unreacted Ti_2_PTe_2_. The lattice parameters of the precursor and the intercalated compounds are shown in Table 1 and the results of elemental analysis by SEM/EDX are shown in Supplementary Figs 5 and 6.

**Figure 1:**
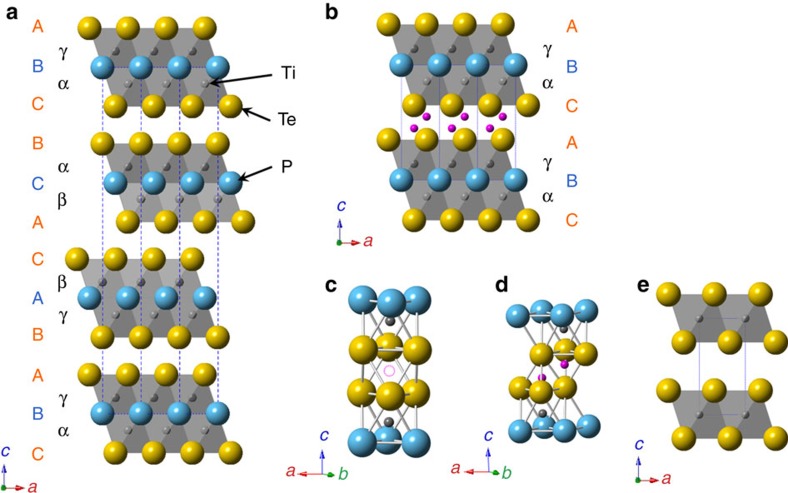
Crystal structures of *T*_2_PTe_2_ (*T*=Ti and Zr) and their intercalated derivative. (**a**) *T*_2_PTe_2_ (3R-Ta_2_CS_2_ type with space group *R*-3*m*) and (**b**) metal-intercalated *M*_*x*_*T*_2_PTe_2_ (1T-Ta_2_CS_2_ type with space group *P*-3*m*1), where black, blue, yellow and purple spheres, respectively denote Ti/Zr, P, Te and *M* atoms. A, B, C are used to represent anion (Te, P) stacking arrangement, while α, β, γ represent the Ti/Zr stacking sequence. The unit cells are shown by the dotted lines. (**c**) Coordination environment around an octahedral void (dotted circle) in the 3R structure. (**d**) Coordination environment around a tetrahedral void in the 1T structure. In *M*_*x*_*T*_2_PTe_2_, the voids are partially occupied by *M* (for example, ∼20% for Zn). (**e**) Crystal structure of TiTe_2_ and *MX*_2_ compounds in general (*M*=Ti, Zr, Ta and so on; *X*=S, Se).

For a more precise structural characterization of Zn_*x*_Ti_2_PTe_2_, synchrotron X-ray diffraction and neutron diffraction Rietveld refinements were carried out. Here, we recall that Ta_2_CS_2_ has two polymorphs with different stacking sequences, the 3R-type (*R*–3*m*) being isostructural with Ti_2_PTe_2_ and the 1T-type (*P*–3*m*1), as displayed in Fig. 1a,b, respectively^33^. Both forms of Ta_2_CS_2_ are capable of accepting various foreign cations, but since the stacking sequences are different, intercalated cations find themselves in different coordination environments. For example, Fe and Co in 3R-*M*_*x*_Ta_2_CS_2_ (*M*=Fe, Co) partially occupy octahedral voids (site 6*c*), whereas Cu in 1T-*M*_*x*_Ta_2_CS_2_ occupies tetrahedral voids (site 2*d*)^33^,^34^,^35^, as illustrated in Supplementary Fig. 7. Given the observed unit cell change in Ti_2_PTe_2_ upon Zn intercalation, it is likely that Zn_*x*_Ti_2_PTe_2_ also adopts the 1T-Cu_*x*_Ta_2_CS_2_ structure. Therefore, this structure was adopted as a starting model for a Rietveld refinement, by placing Zn at the 2*d* (1/3, 2/3, *z*) site (Fig. 1b,d). The synchrotron pattern also shows minor impurities, TiP and ZnTe, which were included in the refinement. The refinements converged comfortably with *R*_wp_=7.51% and *R*_p_=5.20% for synchrotron X-ray diffraction and *R*_wp_=6.36% and *R*_p_=4.95% for neutron diffraction (see Fig. 3a,b and Supplementary Table 1). The refined Zn composition of *x*=0.40 is close to the value obtained from the EDX measurements. Placing Zn at the octahedral 1*b* site (Supplementary Fig. 7) did not lead to better convergence. Likewise, the synchrotron X-ray diffraction refinement for Cu_*x*_Ti_2_PTe_2_, Zn_*x*_Zr_2_PTe_2_ and Cd_*x*_Zr_2_PTe_2_ revealed the occupation of Cu, Zn and Cd at the tetrahedral (2*d*) site and a composition of *x*=0.282(2), 0.337(6) and 0.194(5) (Fig. 3d, Supplementary Fig. 8 and Supplementary Table 2).

For Ti_2_PTe_2_, the refined atomic coordinate *z* for *M* is slightly different between Zn and Cu. The Cu position (*z*=0.452) is nearly at the centre of the Te_4_ tetrahedron. In contrast, the Zn position (*z*=0.4347) is slightly off-centred as found in 1T-Cu_*x*_Ta_2_CS_2_ (ref. 34), giving Zn–Te distances of 2.287 Å ( × 1) and 2.517 Å ( × 3). The former value is shorter than those of common phases such as ZnTe (2.643 Å) and ZnAl_2_Te_4_ (2.578 Å; refs 36, 37). This is rather curious, but a number of techniques support this observation. The charge density distributions around Zn obtained by a MEM analysis shows a strong covalent Zn–Te bond (due to the smaller electronegativity of tellurium), indicative of the single short Zn–Te distance (Fig. 3a). Extended X-ray absorption fine structure analysis at the Zn K-edge (Fig. 3c, Supplementary Fig. 9 and Supplementary Table 3) also led to a better fit with the Rietveld structure incorporating the long/short Zn–Te bonds, as opposed to an isotropic model where all four Zn–Te bonds are the same length. The octahedral model (Zn at the 1*d* site) also failed to reproduce the experimental data. The off-centring of Zn may arise to reduce repulsion from the Ti located on the top of Zn (see Fig. 1d).

**Figure 2:**
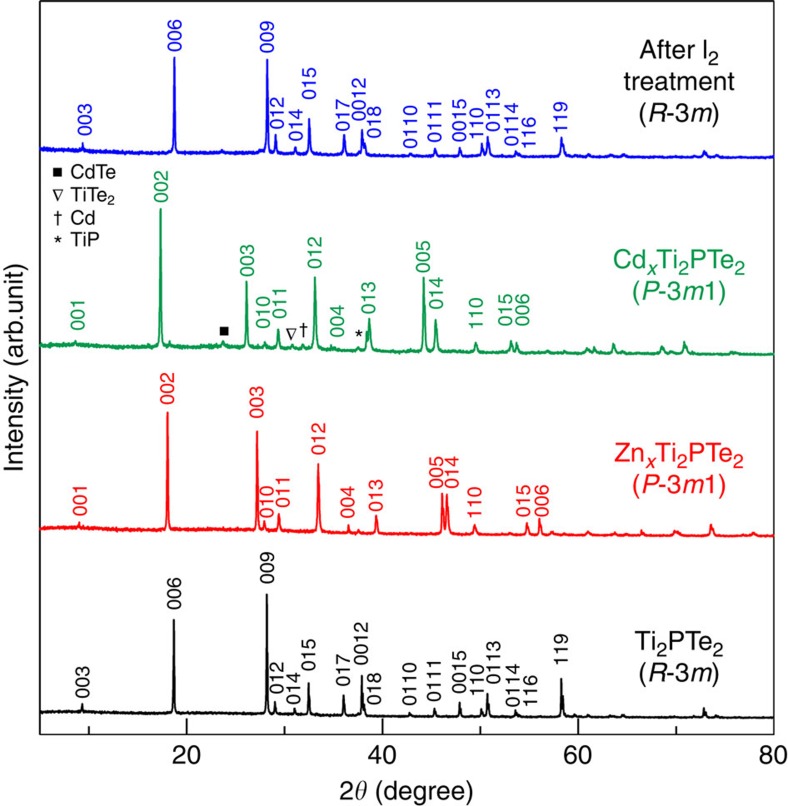
X-ray diffraction patterns of Ti_2_PTe_2_ and (de)intercalated materials. From bottom to top: Ti_2_PTe_2_, Zn_*x*_Ti_2_PTe_2_ (300 °C, 48 h, *p*=0.4), Cd_*x*_Ti_2_PTe_2_ (200 °C, 48 h, *p*=0.4), deintercalated product after I_2_ treatment of Zn_*x*_Ti_2_PTe_2_ at room temperature. Peaks with symbols show tiny amount of impurity phases (CdTe, TiTe_2_ and so on) for Cd intercalation.

**Figure 3:**
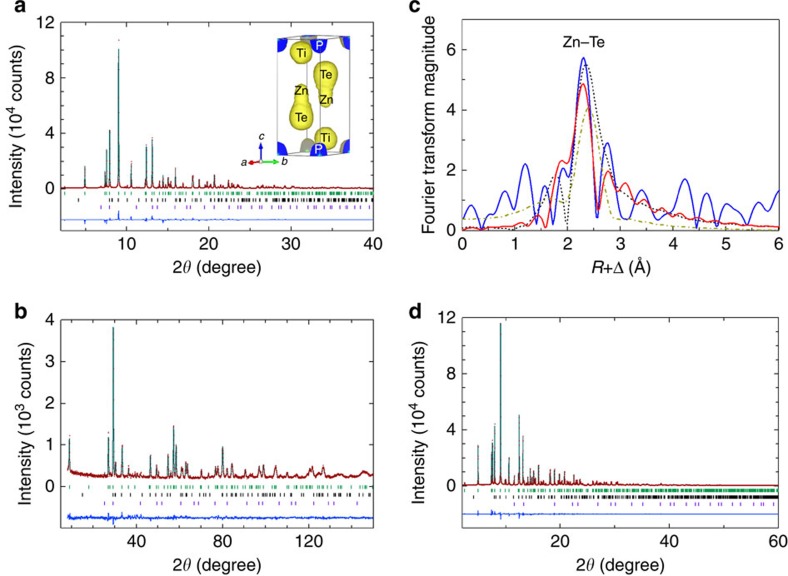
Structural refinement for *M*_*x*_Ti_2_PTe_2_ (*M*=Zn, Cu). Refined (**a**) Synchrotron X-ray diffraction and (**b**) neutron diffraction patterns of Zn_0.4_Ti_2_PTe_2_, showing observed (red), calculated (green) and difference (blue) profiles. The upper, middle and lower ticks represent the positions of the calculated Bragg reflections of Zn_0.4_Ti_2_PTe_2_, TiP and ZnTe, respectively. Inset of **a** shows the result of MEM analysis, where a strong Zn–Te bond along the *c* axis was observed. (**c**) Fourier transforms of the extended X-ray absorption fine structure (EXAFS) spectrum (blue) at the Zn K-edge of Zn_0.4_Ti_2_PTe_2_, which is compared with calculated Fourier transforms of (red) the anisotropic tetrahedron based on the neutron diffraction refined crystal structure, (dotted line) the equidistant tetrahedron and (dashed line) octahedron. (**d**) Refined synchrotron X-ray diffraction pattern of Cu_0.28_Ti_2_PTe_2_. The upper, middle and lower ticks represent the positions of the calculated Bragg reflections of Cu_0.28_Ti_2_PTe_2_, TiP and Cu, respectively.

The reversibility of the intercalation process is demonstrated by regeneration of the original Ti_2_PTe_2_ host upon exposure to I_2_ in acetonitrile at room temperature, as determined by X-ray diffraction (Fig. 2). The I_2_ reaction gives rise to extra tiny peaks such as at 27.8°, which can be attributed to Te metal. This possibly results from the decomposition of ZnTe, a tiny impurity phase already present in Ti_2_PTe_2_. For the initial Zn intercalation, we further examined the reaction products while varying the Zn/Ti_2_PTe_2_ molar ratio *p* (0.1≤*p*≤0.5), with a fixed reaction temperature and time (300 °C, 48 h). For *p*=0.1, the 3R phase coexists with traces of the 1T phase. With increasing *p*, the amount of the latter phase increases, whereas the former decreases, and the lattice constants of the rhombohedral phase do not change (Supplementary Fig. 10). In other words, there is no uptake of guest ions in the original 3R phase and Zn intercalation readily induces structural transition and the phase separation. This has been confirmed by SEM/EDX results (Supplementary Fig. 3).

### Theoretical calculations

As discussed above, upon intercalation 3R-*T*_2_PTe_2_ transforms to the 1T polytype, and metals amenable to intercalation (Cd, Zn, Cu, Fe, Mn for *T*=Ti and Cd, Zn, Cu for *T*=Zr) are less electropositive and favour tetrahedral coordination with Te^38^. However, no intercalation of alkali metals was observed. Such intercalation selectivity in Ti_2_PTe_2_ is quite unprecedented and clearly different from other layer materials based on oxide, sulphide and chloride with weaker interlayer vdW bonding, such as V_2_O_5_, *M*NCl, *MX*_2_ (*X*=S, Se) and *M*P*X*_3_ (*X*=S, Se)^8^,^9^,^10^,^11^. Apparently, the use of less electronegative Te layers (versus O, S, Se and so on) hampers alkali metals intercalation. Interestingly, the related telluride TiTe_2_ is known to accommodate Li and Rb, together with transition metals^28^,^39^,^40^,^41^,^42^. Namely, the intercalation selectivity of TiTe_2_ and ZrTe_2_ can be greatly enhanced by Ti_2_PTe_2_ and Zr_2_PTe_2_. This clear difference in intercalativity may originate from the difference in crystal structure and electronic structure. Compared with the structure of *T*Te_2_ having *T*Te_6_ octahedra (Fig. 1e), *T*_2_PTe_2_ possesses an additional P layer, thus providing an anisotropic octahedral coordination of *T*Te_3_P_3_ (Fig. 1a,b). An additional notable feature in *T*_2_PTe_2_—being distinct from *T*Te_2_ and other intercalation materials with vdW layers—is the presence of one extra electron per formula in the conduction band, which is described by the nominal charge configuration: (*T*^4+^)_2_(P^3−^)(Te^2−^)_2_(e^−^), though this and intercalated compounds are basically covalent in nature. This is evidenced by X-ray absorption near edge structure (XANES) results showing the Ti^4+^ state and fairly good metallic conductivity (*ρ*=40 μΩ) at room temperature^31^.

**Figure 4:**
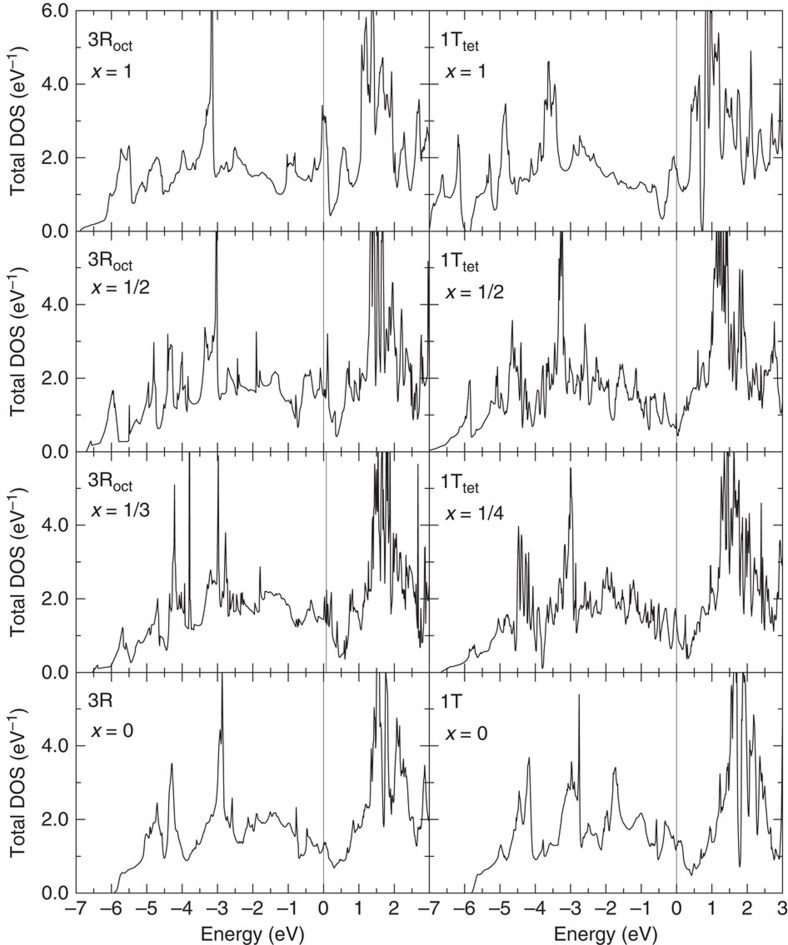
Calculated total density of states of Zn_*x*_Ti_2_PTe_2_ as a function of Zn concentration *x*. Left: 3R-type Zn_*x*_Ti_2_PTe_2_ with octahedral coordinate Zn; and right: 1T-type Zn_*x*_Ti_2_PTe_2_ with tetrahedral coordinate Zn. The Fermi energy is set to the origin of energy.

Calculations based on density functional theory have revealed unique features in the electronic structures and bonding character of this material. The results show that 3R-Ti_2_PTe_2_ is indeed metallic with a nearly half-filled electron Fermi surface, as illustrated in Fig. 4 and Supplementary Fig. 11. This metallic feature is in sharp contrast to TiTe_2_, where hole and electron Fermi surfaces are compensated and a semimetallic-like electronic state is realized^43^. Despite the two-dimensional crystal structure in Ti_2_PTe_2_, the Fermi surface has a three-dimensionally deformed shape (Supplementary Fig. 11b) and the estimated Fermi velocity is *v*_*x*_=2.70 × 10^7^ cm s^−1^ and *v*_*z*_=1.83 × 10^7^ cm s^−1^, giving rise to a rather small anisotropy (*v*_*z*_/*v*_*x*_)=0.68. States around the Fermi energy consist mainly of Ti *d* orbitals hybridized with Te *p* orbitals, as found in the partial density of states (DOS) in Supplementary Fig. 11. Moreover, the sizable Te–Te hybridization accounts for the emergence of the three-dimensionality of the Fermi surface. Interestingly, the calculated DOS for the dummy ‘host' material 1T-Ti_2_PTe_2_ share similar features with those of 3R-Ti_2_PTe_2_, such as a pseudogap structure (valley) just above the Fermi energy (Fig. 4), naively implying that electron doping up to the pseudogap possibly stabilizes the intercalated phases within a rigid band picture.

Total energy calculations for Zn_*x*_Ti_2_PTe_2_ with different Zn concentrations (0≤*x*≤1), intercalation sites (tetrahedral or octahedral) and ‘host' structures (3R or 1T) provide useful information on the phase stability of the present system (see Fig. 4). Calculated heats of formation *E*_f_=*E*(1T-Zn_*x*_Ti_2_PTe_2_)−*E*(3R-Ti_2_PTe_2_)−*xE*(Zn) in the 1T phase show very small positive values (in the order of 10 meV) up to *x*=0.5, implying that an entropic term may stabilize the telluride upon intercalation of Zn. This dopant level is fairly consistent with the experimentally obtained value of ∼0.4. Further electron doping beyond the pseudogap results in the appearance of a peak around the Fermi energy (see the *x*=1 case in Fig. 4), indicating non-bonding electrons. On the other hand, the Zn intercalation in the 3R phase gives rise to a much larger 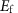 (in the order of 100 meV) even with a small dopant concentration such as *x*=1/3, and the DOS exhibit a peak structure around the Fermi energy as shown in Fig. 4, indicative of the deviation from the rigid band picture. The DOS peak is composed mainly of Ti-*d* and Zn-*s* orbitals, preventing the Zn intercalation. As for Zn_*x*_Zr_2_PTe_2_, qualitative features in the calculated electronic structure are quite similar to Zn_*x*_Ti_2_PTe_2_, showing even smaller (still positive) heats of formation in the 1T phase.

**Figure 5:**
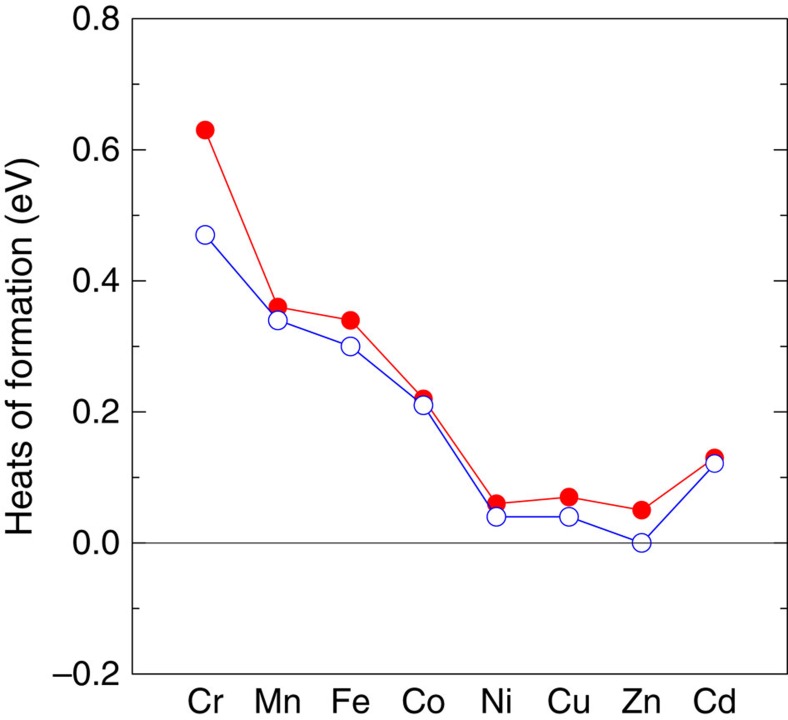
Calculated heats of formation per formula unit in *M*_0.25_*T*_2_PTe_2_ (*T*=Ti, Zr). The heat of formation was defined as *E*_f_=*E*(1T-*M*_0.25_*T*_2_PTe_2_)−*E*(3R-*T*_2_PTe_2_)−0.25*E*(*M*), where *T*=Ti (red) and Zr (blue); *M*=Cr, Mn, Fe, Co, Ni, Cu, Zn and Cd.

To obtain further insight into the metal selectivity, we performed the first principles calculations for 1T-*M*_0.25_Ti_2_PTe_2_ (*M*=Cr, Mn, Fe, Co, Ni, Cu, Zn and Cd) and 1T-*M*_0.25_Zr_2_PTe_2_ (*M*=Ni, Cu, Zn and Cd) with *M* sitting on the tetrahedral interstitial site, and estimated the heat of formation, defined as *E*_f_=*E*(1T-*M*_0.25_Ti_2_PTe_2_)−*E*(3R-Ti_2_PTe_2_)−0.25*E*(*M*). As shown in Fig. 5, a positive but very small value of *E*_f_ for Zn intercalated both in Ti_2_PTe_2_ and Zr_2_PTe_2_ is clearly seen, while marginally small values are achieved for *M*=Ni, Cu and Cd compared with the intercalated systems with *M*=Cr, Mn, Fe and Co, which reasonably confirms the experimentally observed metal selectivity. These results demonstrate that the thermodynamic stability of the intercalated compound is the decisive factor in determining the selectivity. We note that unintercalated Ni has similarly small values of *E*_f_=+0.06 eV (Ti_2_PTe_2_) and +0.04 eV (Zr_2_PTe_2_), the reason of which is not clear but may be related to kinetic aspects of reactions (diffusion of metal, surface reactions and so on).

### Towards practical reaction conditions

**Figure 6:**
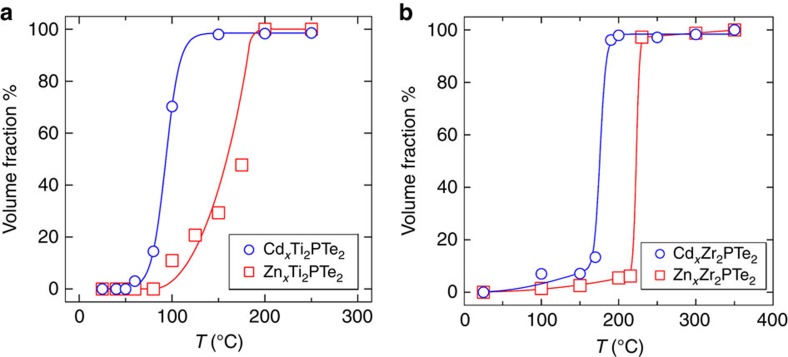
Temperature-dependent intercalation yields for Ti_2_PTe_2_ and Zr_2_PTe_2_. Reaction temperature dependence of the volume fraction of (**a**) *M*_*x*_Ti_2_PTe_2_ and (**b**) *M*_*x*_Zr_2_PTe_2_ where *M*=Zn (red) and Cd (blue). Both the samples were heated together with and elemental metal (Zn or Cd) at various temperatures for 48 h. The molar volume fraction was estimated from the Rietveld refinement. Solid lines are drawn as a guide for visualization. The error bars are smaller than the size of the symbols.

A remarkable aspect in Ti_2_PTe_2_ and Zr_2_PTe_2_ is the facile uptake of heavy metals at mild temperatures and in the solid state. We tested the effect of reaction temperature for *M*=Zn and Cd, with a fixed reaction time of 48 h. Shown in Fig. 6 and Supplementary Fig. 12 is the fraction of the intercalated (1T) phase as a function of reaction temperature. In the case of Ti_2_PTe_2_, the volume fraction of Cd-intercalated phase readily increases at temperatures above 50 °C and reaches almost 100% at 150 °C. In contrast, a higher temperature of 200 °C is necessary to attain a full uptake of Zn. The Zr_2_PTe_2_ system has quantitatively a similar tendency in intercalation behaviour, but the required temperatures, 190 °C (Cd) and 220 °C (Zn), are higher than those in Ti_2_PTe_2_. In Ti_2_PTe_2_, Cu requires 300 °C and Fe and Mn requires >400 °C, given that the same reaction period of 48 h is applied. Furthermore, the reaction of Zr_2_PTe_2_ with Cu at 70 °C for 20 days yielded a single phase of 1T-Cu_*x*_Zr_2_PTe_2_ (Supplementary Fig. 13). These results suggest that this type of intercalation could be further expanded, with a versatile potential to control the reactivity and selectivity.

## Discussion

The difference in the intercalation temperature makes Ti_2_PTe_2_ and Zr_2_PTe_2_ of interest as adsorbents to chemically separate Cd from other metals simply by tailoring the temperature. In many forms, Cd has been (or was) widely used and discharged from the electroplating industry, electrical contact devices, nickel cadmium batteries and pigments^44^,^45^. Traditional metal separation procedures (smelting, precipitation, ion exchange and solvent extraction) are solution-based^46^. As shown in Supplementary Figs 14 and 15, test reactions of Ti_2_PTe_2_ with an equimolar mixture of Cd-Mn at 100 °C for 96 h and Cd-Ti at 300 °C for 48 h yielded only the Cd intercalated material. Selective intercalation of Cd-containing alloys might also be possible. Reactions with commercially available alloys, Cu_0.75_Pb_0.25_ and Cu_0.80_Sn_0.20_, at 300 °C, 48 h resulted in only Cu accommodation into Ti_2_PTe_2_ as shown in Supplementary Fig. 16. The present solution-free approach may find a new route for capturing Cd and other toxic elements. It is noteworthy that the vacuum environment is not a requirement; reactions in ambient conditions yielded the same result (see Supplementary Fig. 17). What we showed here is a prototype application in environmental remediation. Given the fact that all the current technologies for metal capturing are based on solution chemistry, our demonstration of the selective solid-state metal capturing (in particular, the Cd capturing above 80 °C for Ti_2_PTe_2_) is the first of its kind in inorganic layered materials, opening new possibilities for applications to solve environmental issues.

In addition, the observed reactivity and highly mobile nature of the heavy metals at very low temperatures suggests a possibility to develop multivalent ion conductors at significantly lowered working temperatures. This compound represents a member of a larger structure family as in Ta_2_CS_2_, with possibilities for improved performance as well as new properties. We believe that layered compounds with less electronegative anions such as telluride and antimonide could provide fertile ground for the development of exotic functionalities.

## Methods

### Materials synthesis

The stoichiometric polycrystalline samples of the host material Ti_2_PTe_2_ and Zr_2_PTe_2_ were prepared by the conventional solid state reaction method with excess P to compensate its loss due to sublimation above 500 °C. Ti/Zr (Kojundo Chemical, 3N), P (Kojundo Chemical, 2N) and Te (Kojundo Chemical, 3N) powders were mixed in the molar ratio of 2: 1.1: 2 and pelletized in a nitrogen-filled glove box. The obtained pellet was sealed into an evacuated silica tube (<10^−2^ Pa) and heated to 850 °C at a rate of 20 °C h^−1^, and annealed for 24 h. To ensure full incorporation of both phosphorus and tellurium, a slow cooling rate of 2 °C h^−1^ was applied from 850 to 400 °C before rapid cooling to room temperature.

The low temperature reactions of Ti_2_PTe_2_ and Zr_2_PTe_2_ with various elemental metals (*M*=Li, Na, Mg, Al, Si, K, Ca, Sc, Ti, V, Cr, Mn, Fe, Co, Ni, Cu, Zn, Ga, Ge, Sr, Y, Zr, Nb, Mo, Ru, Pd, Ag, Cd, In, Sn, Hf, Ta, Ir, Hg, Pb) were performed in the solid state. Ti_2_PTe_2_ and the metal powder as received (Kojundo Chemical, 4N) were mixed with various molar ratios (typically 1: 1) and pelletized in a nitrogen-filled glove box. The pellet was sealed in an evacuated silica tube (<10^−2^ Pa) and heated at temperatures between 100 °C and 400 °C for 48–96 h. Reactions with equimolar mixtures of Cd and Ti and of Cd and Mn in Ti_2_PTe_2_ were conducted, respectively, at 300 °C for 48 h, and at 100 °C for 96 h. Also, reactions with Cu_0.75_Pb_0.25_ alloy and with Cu_0.80_Sn_0.20_ alloy (Kojundo Chemical, 3N) were performed at 300 °C for 48 h. Furthermore, for lithium intercalation, 1 M *n*-butyl lithium solution in hexane was used and the reaction allowed to proceed for 72 h. Deintercalation of Zn was performed at room temperature by mixing Zn_0.4_Ti_2_PTe_2_ with I_2_ dissolved in acetonitrile at the molar ratio of 1: 1 for 2 days.

### X-ray and neutron diffraction

Laboratory powder X-ray diffraction were collected using a Bruker D8 diffractometer with Cu *K*_α_ radiation. The diffraction data for structural refinement were recorded in a 2*θ* range from 5° to 80° with a step interval of 0.02°. The diffraction pattern for Ti_2_PTe_2_ was fit using the space group *R*–3*m* and the cell parameters *a*=3.63949(6) Å and *c*=28.4885(4) Å, in agreement with reported values in the literature^27^. High resolution synchrotron X-ray diffraction experiments were performed on Zn_*x*_Ti_2_PTe_2_, Cu_*x*_Ti_2_PTe_2_, Zn_*x*_Zr_2_PTe_2_ and Cd_*x*_Zr_2_PTe_2_ at room temperature on a Debye-Scherrer camera installed at beamline BL02B2, SPring-8. The incident beam from a bending magnet was monochromatized to 0.35479(1) Å. The powder samples were loaded into a glass capillary (0.1 mm inner diameter) and rotated during measurements to reduce preferential orientation. The diffraction data were recorded in a 2*θ* range from 0° to 60° with a step interval of 0.01°. Powder neutron diffraction measurements were carried out at room temperature on an approximately 2 g sample at BT-1 (*λ*=1.5403 Å), National Institute of Standards and Technology.

### Structural analysis

The obtained X-ray and neutron data were analysed by the Rietveld method using the RIETAN-FP program^47^. The agreement indices used were *R*_p_=Σ|*y*_io_−*y*_ic_|/Σ*y*_io_, *R*_wp_=[Σ*w*_i_(*y*_io_−*y*_ic_)^2^/Σ*w*_i_(*y*_io_)^2^]^1/2^ and the goodness of fit, *χ*^2^=[*R*_wp_/*R*_exp_]^2^ where *R*_exp_=[(*N*−*P*)/Σ*w*_i_*y*_io_^2^]^1/2^, *y*_io_ and *y*_ic_ are the observed and calculated intensities, *w*_i_ is the weighting factor, *N* is the total number of *y*_io_ data when the background is refined and *P* is the number of adjusted parameters. The energy dispersive X-ray spectroscopy (EDX) measurements were performed using an Oxford Instruments IE-250 detector attached to a scanning electron microscope (SEM, HITACHI S-3400N). For each composition, 10–40 randomly selected spots were examined.

For X-ray absorption spectroscopy (XAS) measurements, Zn_0.4_Ti_2_PTe_2_ was homogeneously dispersed in dried boron nitride powder and pelletized. Zn *K*-edge spectra were recorded in transmission mode at beam line BL01B1 in SPring-8, Japan with a double-crystal Si(111) monochrometer. The energy scale was calibrated using Cu foil. Data were collected at room temperature. Extended X-ray absorption fine structure (EXAFS) analysis was performed using REX2000 data analysis software, with the theoretical backscattering phases and amplitudes calculated with the code FEFF8 (ref. 48). Radial structure functions were obtained using Fourier transformation of the oscillations between 3.0 and 14.0 Å. To obtain local structural parameters, inverse Fourier transforms were calculated from the radial structure functions between 1.688 and 2.700 Å. Curve fitting was performed in *k* space.

### First principles calculations

First principles density functional theory calculations are performed with all-electron full-potential linearized augmented plane wave method in the scalar-relativistic scheme. Fractional intercalation is simulated by assuming super cell models appropriate to given concentrations. Lattice constants and internal atomic positions are fully optimized by calculating total energy and atomic forces with preserving the original crystal symmetry.

### Data availability

The data that support the findings of this study are available from the corresponding author upon request.

## Additional information

**How to cite this article:** Yajima, T. *et al*. Selective and low temperature transition metal intercalation in layered tellurides. *Nat. Commun.* **7,** 13809 doi: 10.1038/ncomms13809 (2016).

**Publisher's note**: Springer Nature remains neutral with regard to jurisdictional claims in published maps and institutional affiliations.

## Supplementary Material

Supplementary Information Supplementary figures and supplementary tables

## Figures and Tables

**Table 1:** Lattice parameters of *T*_2_PTe_2_ (*T*=Ti and Zr) and metal-intercalated *M*_*x*_*T*_2_PTe_2_.

*T*	Ti		Zr	
*M*	*a* (Å)	*c* (Å)	*a* (Å)	*c* (Å)
Parent phase	3.6393 (2)	**9.4951 (4)	3.8142 (3)	**9.7271 (6)
Zn	3.6917 (4)	9.8480 (7)	3.85873 (7)	10.1664 (1)
Cu	3.6726 (4)	9.7822 (7)	3.8445 (2)	10.1082 (5)
Cd	3.6806 (6)	10.238 (1)	3.8469 (2)	10.4197 (4)
*Fe	3.6356 (4)	9.639 (1)	NA	NA
*Mn	3.648 (2)	9.628 (4)	NA	NA

The values are received from the LeBail analysis of laboratory synchrotron X-ray diffraction patterns which are shown in Fig. 2, Supplementary Figs 2 and 4. Fe_*x*_Ti_2_PTe_2_ and Mn_*x*_Ti_2_PTe_2_ were yielded together with a large amount of unreacted Ti_2_PTe_2_. **For *T*_2_PTe_2_ with the 3R structure, the normalized *c* constant (*c*/3) is shown for the sake of comparison. NA, not available.
