# Supplementary material for: Selective and low temperature transition metal intercalation in layered tellurides
Source: Nat Commun. 2016 Dec 14;7:13809. doi: 10.1038/ncomms13809 (PMC5171714; doi:10.1038/ncomms13809)
Supplement: Supplementary Information — Supplementary figures and supplementary tables [file ncomms13809-s1.pdf]

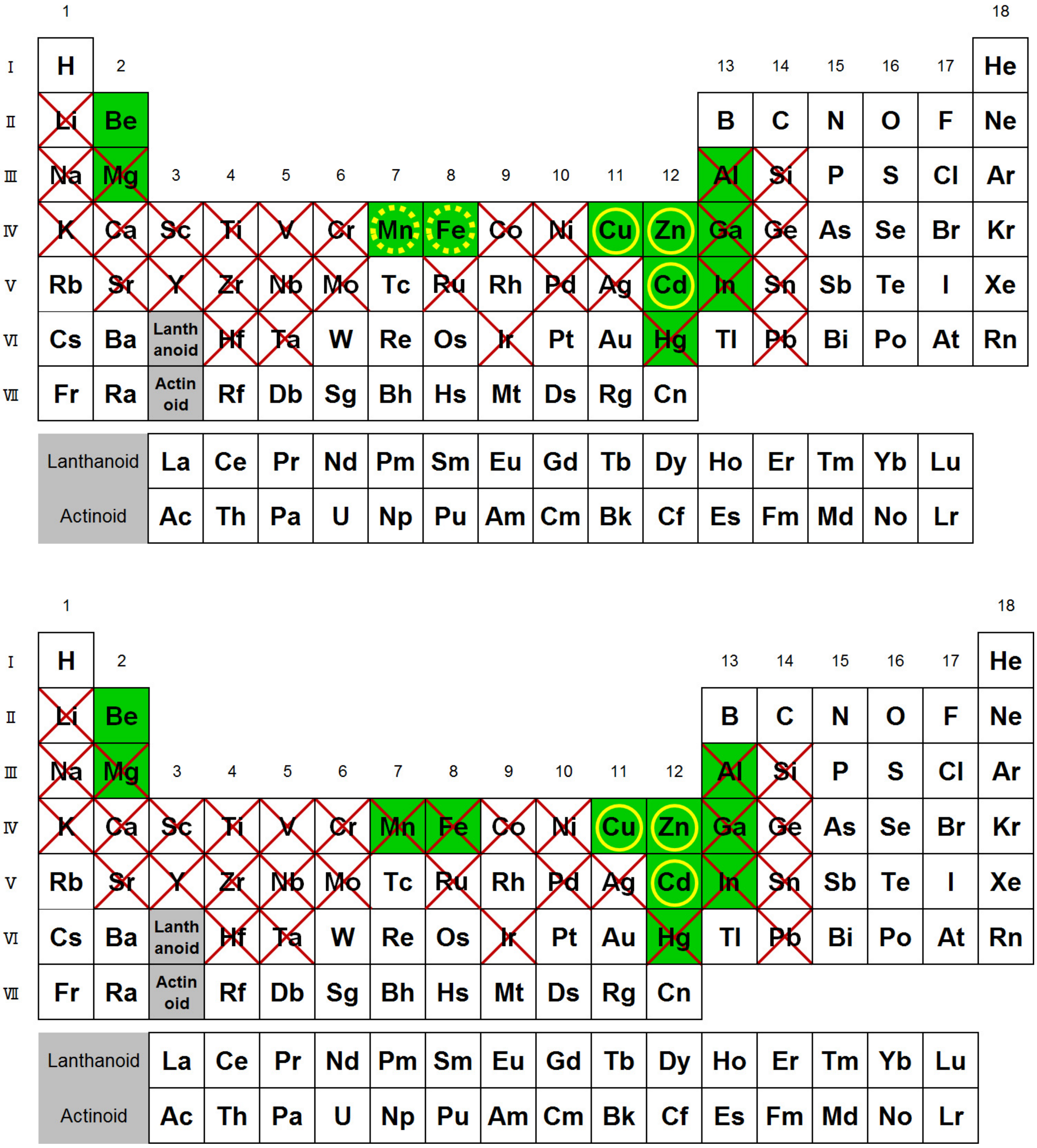

Supplementary Figure 1. Summary of the intercalation reactions with Ti_2PTe_2 (upper) and Zr_2PTe_2 (lower)**.** Elements marked with circles and crosses, respectively denote successful and failed intercalation reactions. Elements showing partial success in intercalation are marked with dotted circles. Coloured green represent the elements that are tetrahedrally coordinated with Te in binary tellurides.

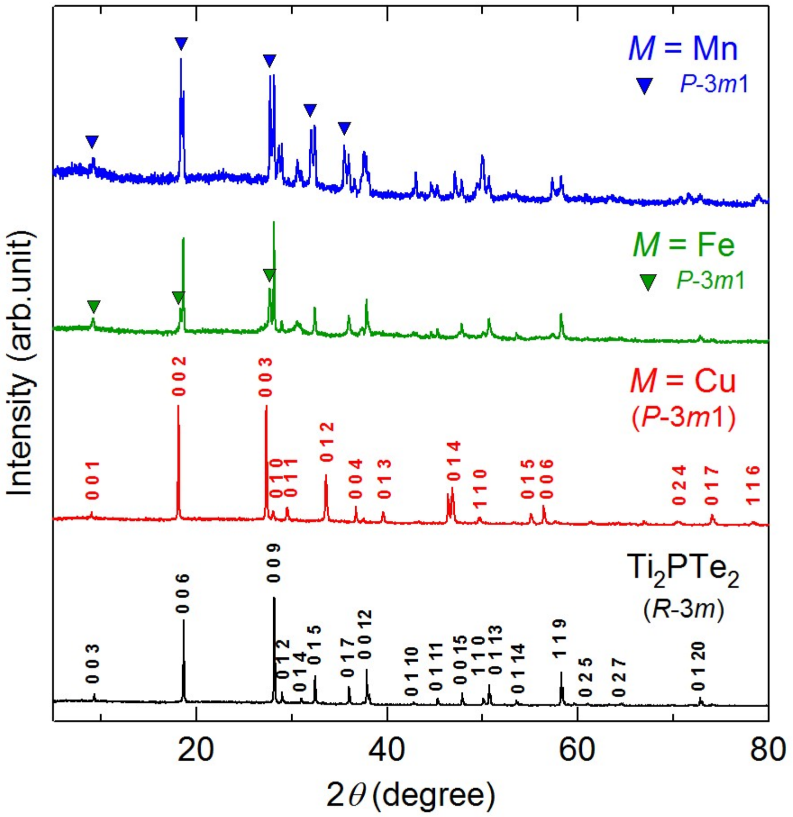

Supplementary Figure 2. XRD patterns of intercalation compounds *M*$_x$Ti$_2$PTe$_2$. (From bottom to top) precursor 3R-Ti$_2$PTe$_2$, 1T-Cu$_x$Ti$_2$PTe$_2$ (reacted with Cu at 300 °C for 48 h), 1T-Fe$_x$Ti$_2$PTe$_2$ (reacted with Fe at 400 °C for 48 h), and 1T-Mn$_x$Ti$_2$PTe$_2$ (reacted with Fe at 400 °C for 48 h). XRD patterns for *M* = Fe and Mn also contain the unreacted 3R-Ti$_2$PTe$_2$ phase.

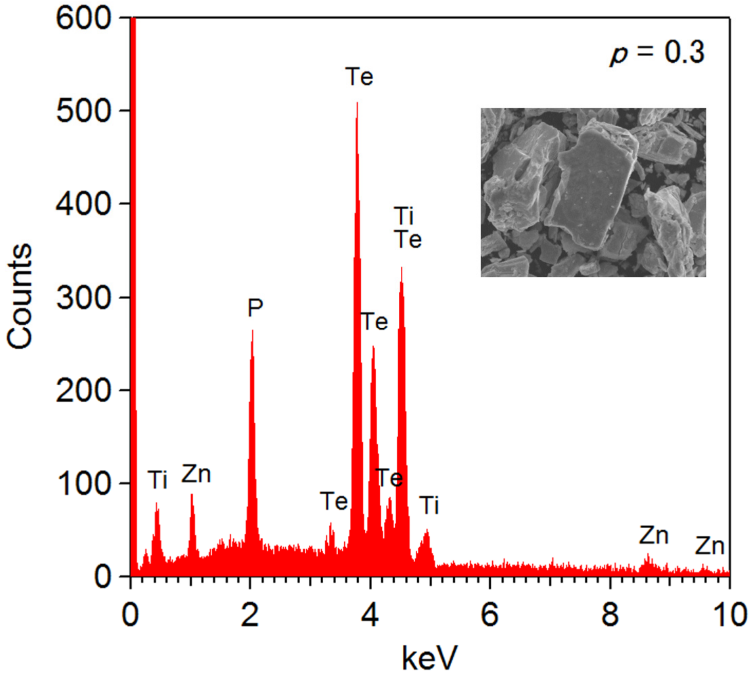

Supplementary Figure 3. Elemental analysis for $Zn_xTi_2PTe_2$. EDX spectrum of the Zn intercalated material ($Zn_xTi_2PTe_2$), obtained at 300 °C for 48 h in vacuum with $p = 0.3$. The SEM image is shown in the inset. This spectrum gives the molar ratio of Zn : Ti : P : Te = 0.37 : 2 : 0.95 : 2.04. We found two types of particles, one without any trace of Zn and the other with a sizable amount of Zn. The former is ascribed to non-intercalated 3R-type Ti_2PTe_2 (unreacted precursor) and the latter to intercalated 1T-type $Zn_xTi_2PTe_2$. As p or the reaction time is increased, fewer non-intercalated particles were observed. It was also found that there is a certain (but not large) degree of compositional (Zn) distributions. Inspection of 25 specimens gave the Zn content of $x = 0.36(5)$.

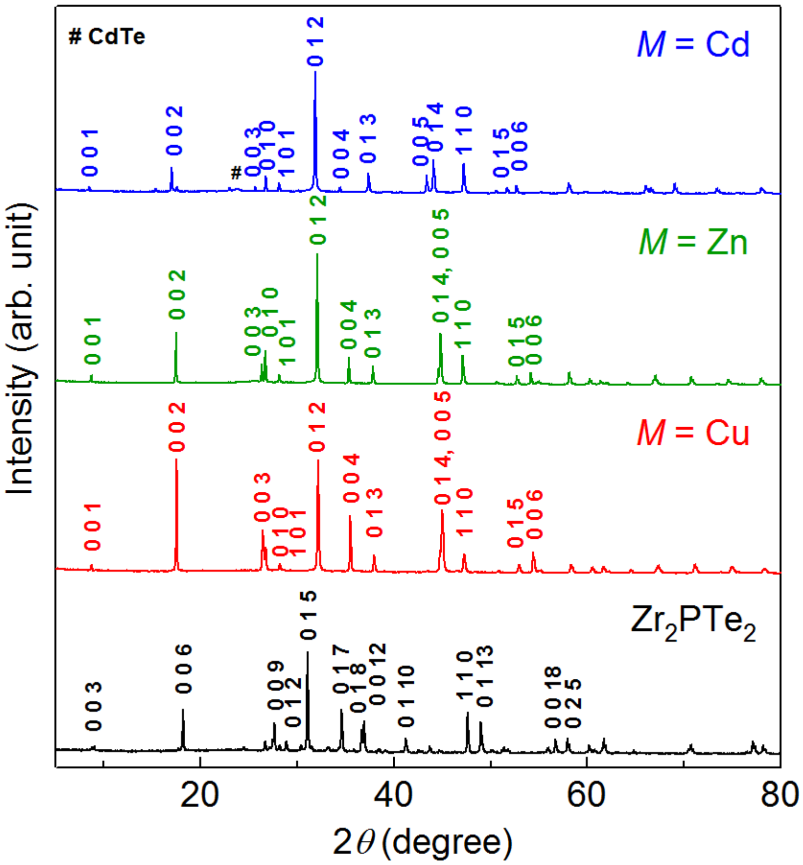

Supplementary Figure 4. XRD patterns of intercalation compounds M_xZr$_2$PTe$_2$. (From bottom to top) precursor 3R-Zr$_2$PTe$_2$, 1T-Cu$_x$Zr$_2$PTe$_2$ (reacted with Cu at 300 °C for 48 h), 1T-Zn$_x$Zr$_2$PTe$_2$ (reacted with Zn at 300 °C for 48 h), and 1T-Cd$_x$Zr$_2$PTe$_2$ (reacted with Cd at 300 °C for 48 h). A small amount of CdTe was observed for Cd intercalation.

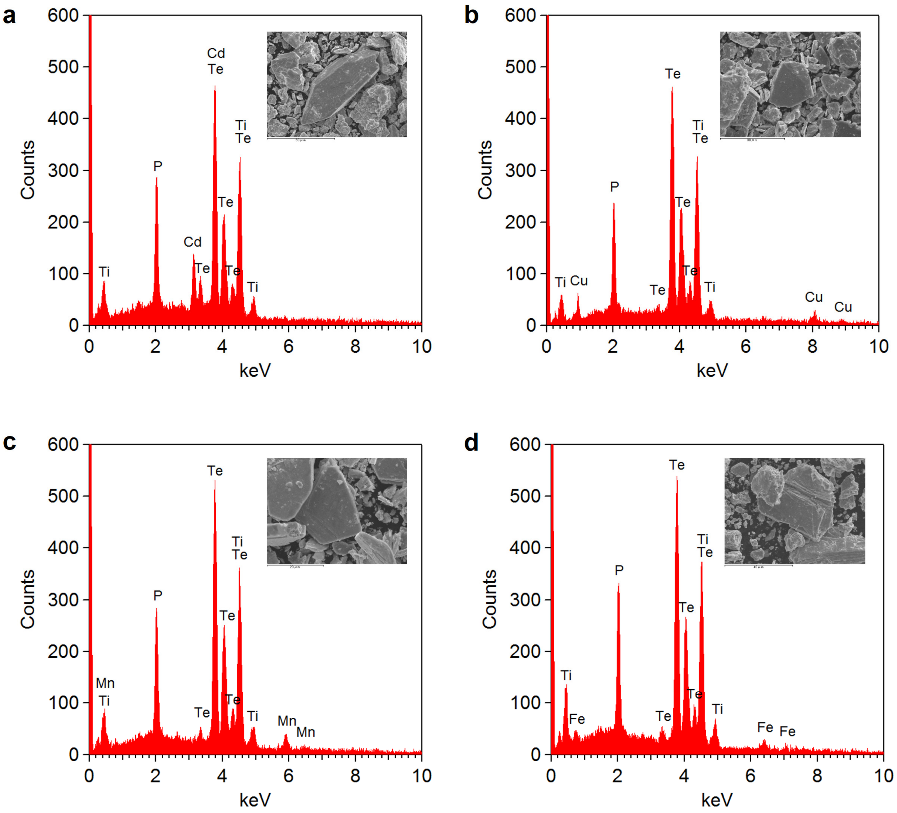

Supplementary Figure 5. EDX spectra for M = (a) Cd, (b) Cu, (c) Mn, and (d) Fe-intercalated $M_x\mathrm{Ti_2PTe_2}$ specimens. Reaction temperature, reaction time and the $M/\mathrm{Ti_2PTe_2}$ ratio (p) are 250 °C, 48 h and p = 1 for Cd, 300 °C, 48 h and p = 0.4 for Cu, 400 °C, 48 h and p = 1 for Fe, 400 °C, 48 h and p = 1 for Mn. These EDX spectra yielded approximate metal contents of x = 0.40, 0.35, 0.15, 0.23 for Cd, Cu, Fe, and Mn.

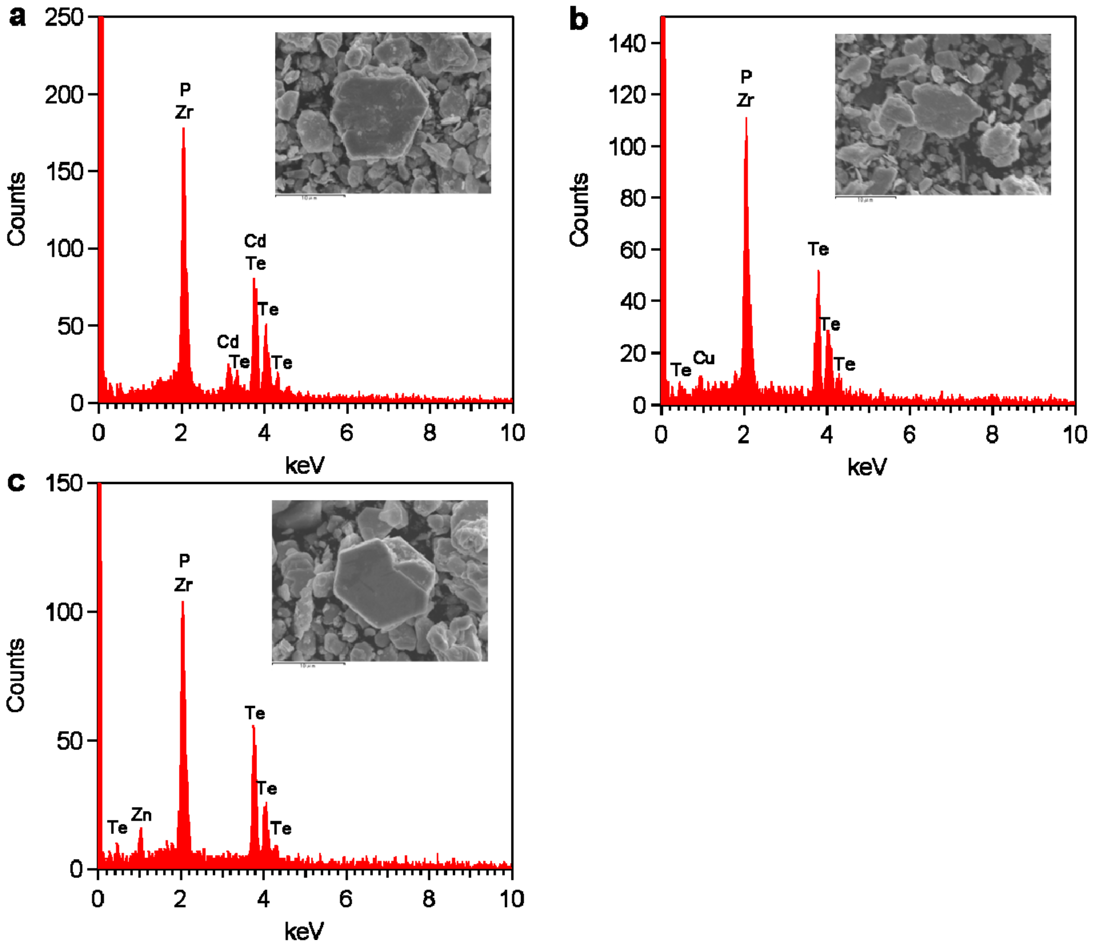

Supplementary Figure 6. EDX spectra for M = (a) Cd, (b) Cu, and (c) Zn-intercalated Zr_2PTe_2 specimens. Reaction temperature, reaction time and the *M*/Zr_2PTe_2 ratio (= *p*) are 300 °C, 48 h and *p* = 1 for Cd, 350 °C, 48 h and *p* = 1 for Cu, 300 °C, 48 h and *p* = 1 for Zn. These EDX spectra yielded approximate metal contents of *x* = 0.36, 0.38, 0.39 for Cd, Cu, and Zn.

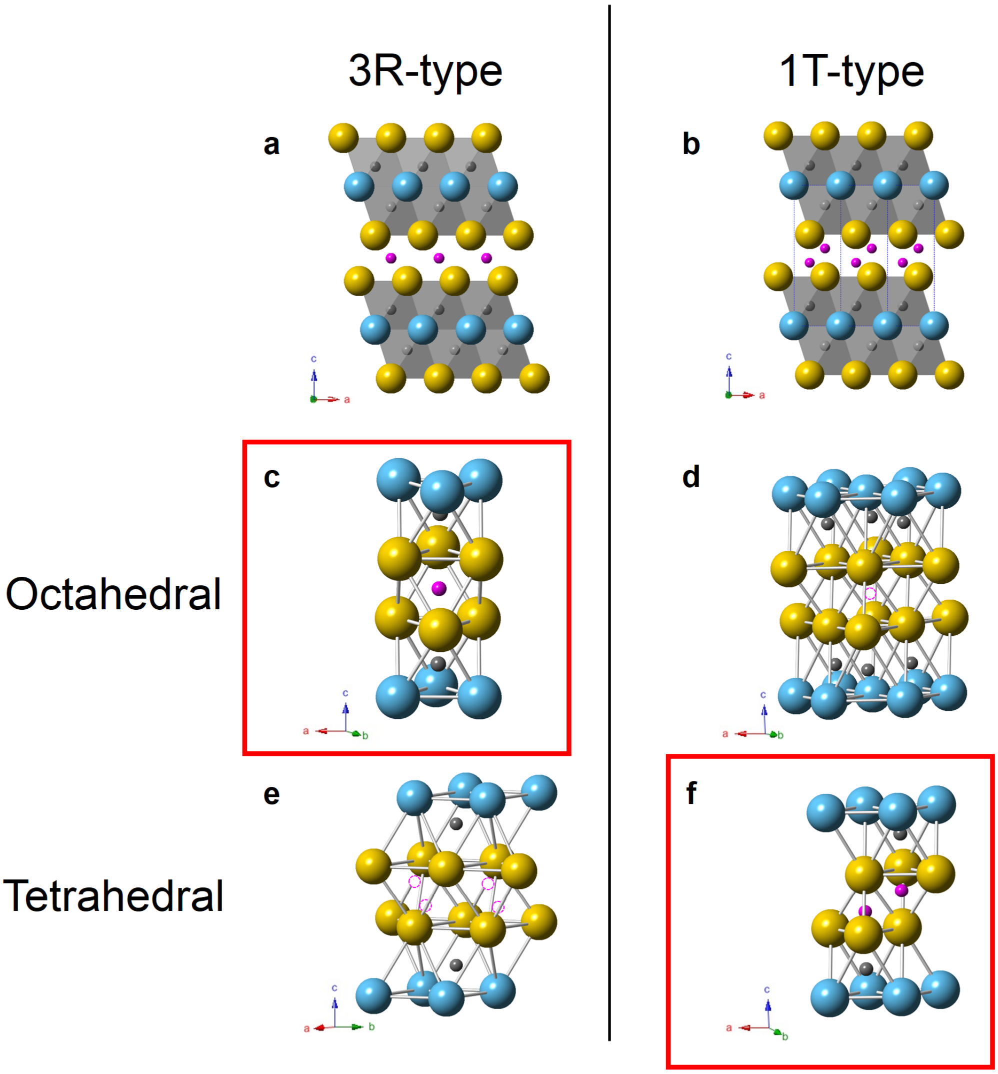

Supplementary Figure 7. Comparison between metal-intercalated 3R-Ta_2CS_2 and 1T-Ta_2CS_2 structures and the coordination environments around the guest metal. a, 3R-type $M_xTa_2CS_2$ structure (R–3m), where black, blue, yellow, and pink spheres represent Ti, P, Te, and M atom, respectively. **b,** 1T-type $M_xTa_2CS_2$ structure (P–3m1). In 3R-type $M_xTa_2CS_2$, M is exclusively coordinated octahedrally (3b) by tellurium atoms (enlarged view in **c**), while in 1T-type $M_xTa_2CS_2$ M is exclusively coordinated tetrahedrally (2d) (enlarged view in **f**).[31] Octahedral voids (1b) in 1T-type structure and tetrahedral voids (18h) for 3R-type Ta_2CS_2 and shown in **d** and **e**. It is known that these voids do

not accommodate any metal. The position of Ti is likely to be relevant for the structural stability; the *M* atoms are located on top of the Ti atom for **c** and **f**, but the voids in **d** and **e** are not. Although Ta_2CS_2 adopts both 3R (**a**) and 1T (**b**) structures, Ti_2PTe_2 and Zr_2PTe_2 can adopt only the 3R structure. Intercalation reactions of Ti_2PTe_2 and Zr_2PTe_2 drive a phase transition to the 1T structure with metals in tetrahedral voids.

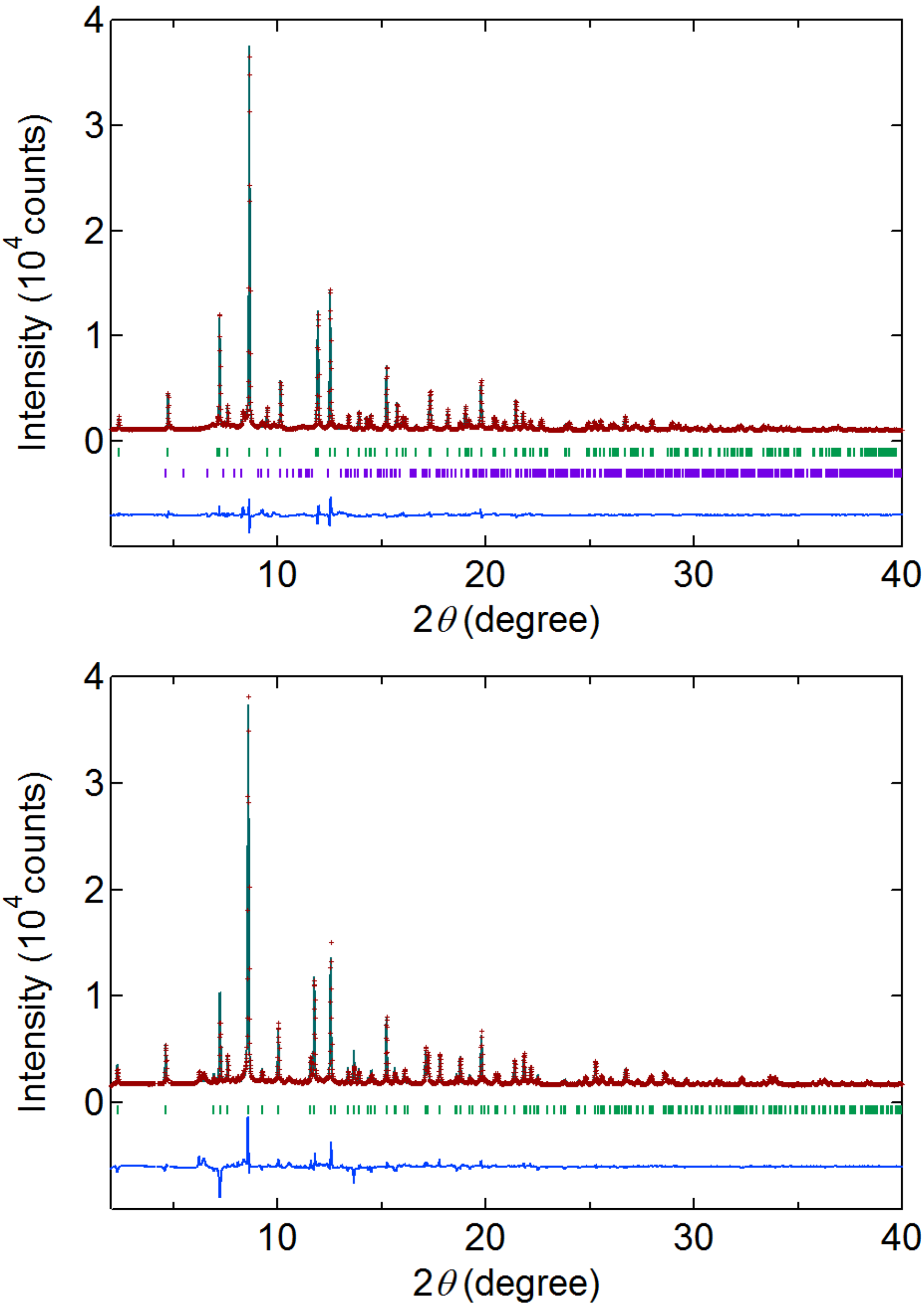

Supplementary Figure 8. Structural refinement for M_xZr$_2$PTe$_2$ (M = Zn, Cd). Refined SXRD patterns of Zn$_{0.34}$Zr$_2$PTe$_2$ (upper) and Cd$_{0.19}$Zr$_2$PTe$_2$ (lower) showing observed (red), calculated (green), and difference (blue) profiles. The ticks represent the positions of the calculated Bragg reflections of M_xZr$_2$PTe$_2$ (green) and ZrP$_2$ (purple).

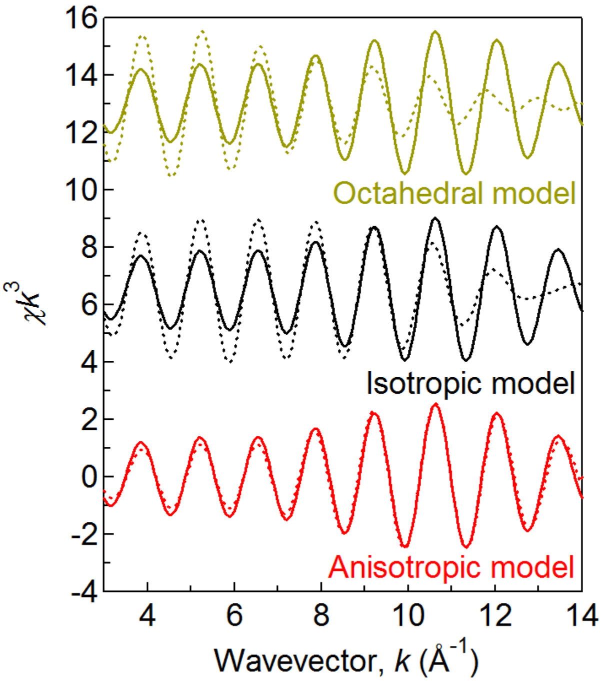

Supplementary Figure 9. Results of curve fitting for the inverse Fourier transform spectra of Zn *K*-edge EXAFS of $Zn_{0.4}Ti_2PTe_2$. The experimental data (solid curve) is compared with the fitting simulations (dotted lines) based on the anisotropic tetrahedral model (bottom), the isotropic (equidistant) tetrahedral model (middle) and the octahedral model (top, also see Supplementary Table 2). It is clear that the anisotropic tetrahedral model gives the best fit.

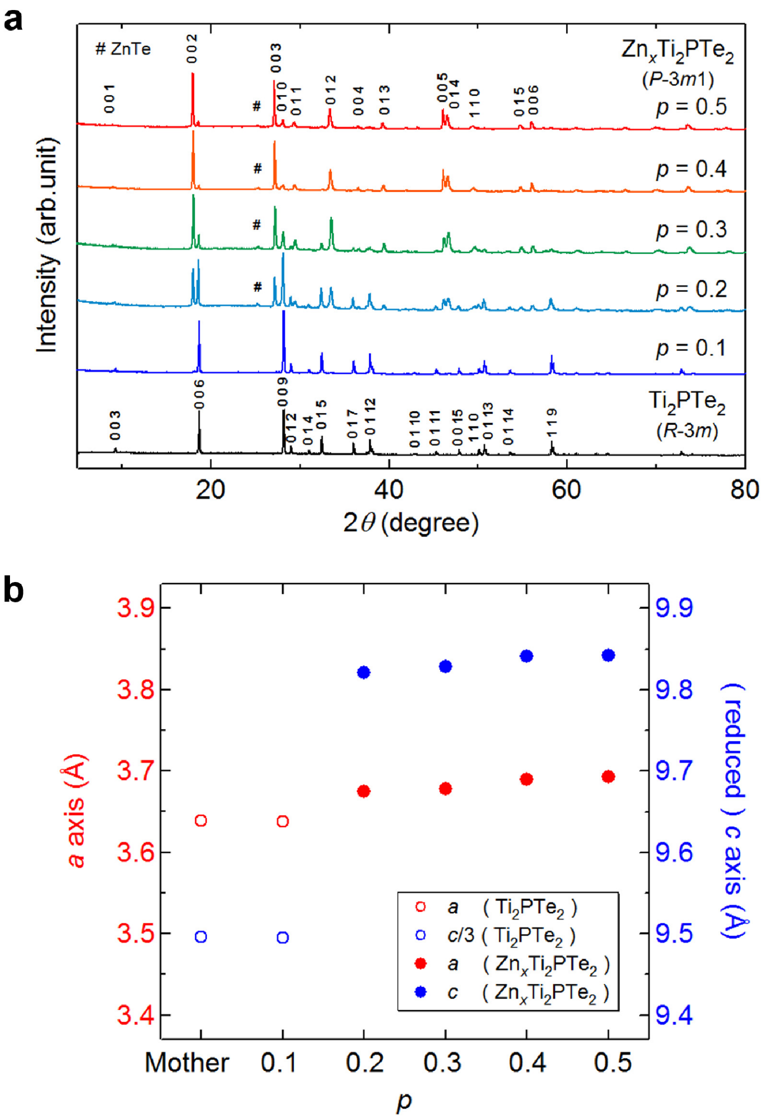

Supplementary Figure 10. Zinc intercalation reacted with various Zn/Ti_2PTe_2 ratios. a, XRD patterns for Zn intercalated materials, prepared at 300 °C for 48 h with various molar ratios *p* (*p* = Zn/Ti_2PTe_2). It is seen that with increasing p, peaks corresponding to the Zn-intercalated 1T phase increase in intensity relative to those of the precursor. **b,** Lattice constants as function of *p*, where for comparison, the normalized *c* parameter (i.e., *c*/3) is plotted for the 3R (non-intercalated) phase. Error bars fit within the markers when plotted.

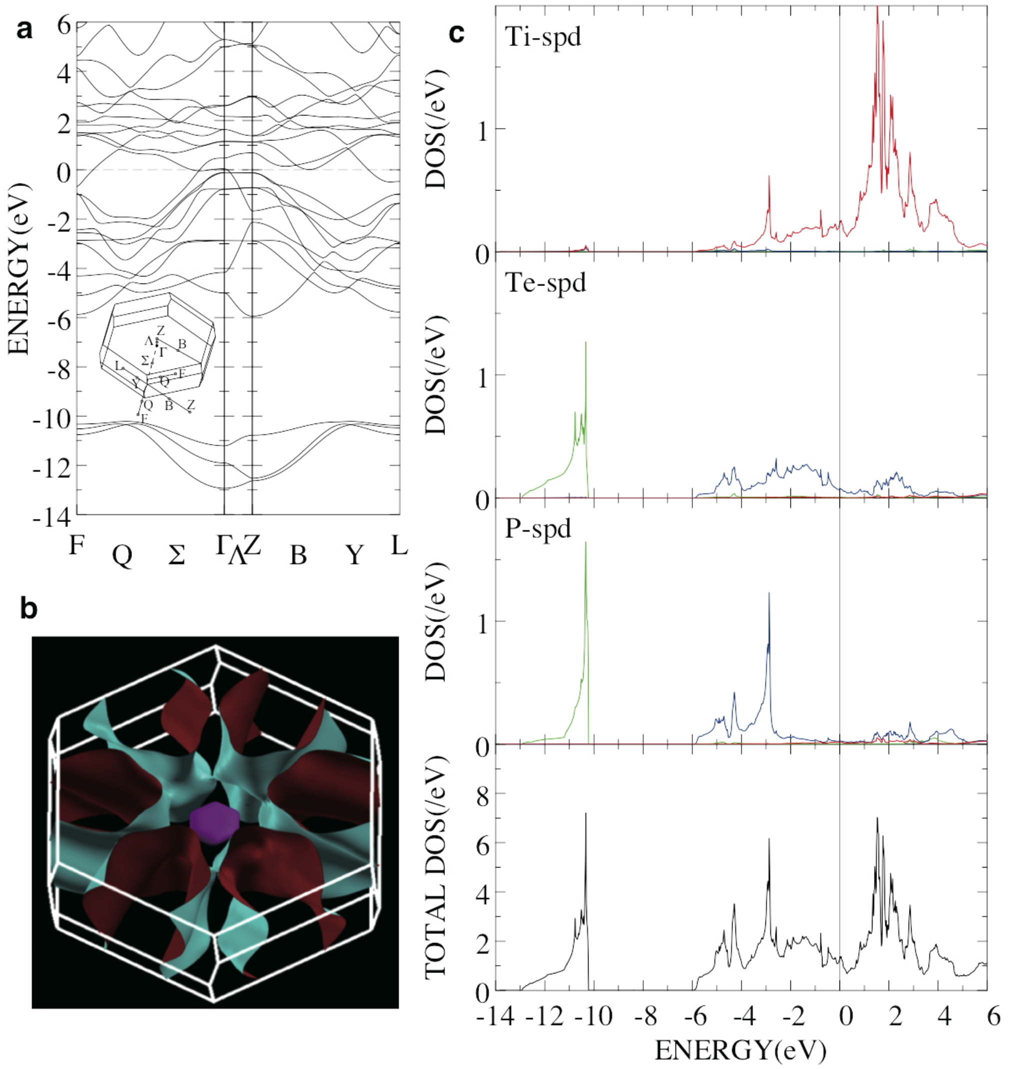

Supplementary Figure 11. Calculated electronic structure of the host Ti_2PTe_2 with the 3R structure. a, Electronic band structure plotted along the high symmetry lines of the rhombohedral Brillouin zone (inset). **b,** A nearly half-filled electron Fermi surface and small hole Fermi surface around Γ. **c,** Total and angular momentum-projected partial density of states (DOS). Green, blue, and red lines denote *s*, *p*, and *d* components of the partial DOS within each muffin tin sphere.

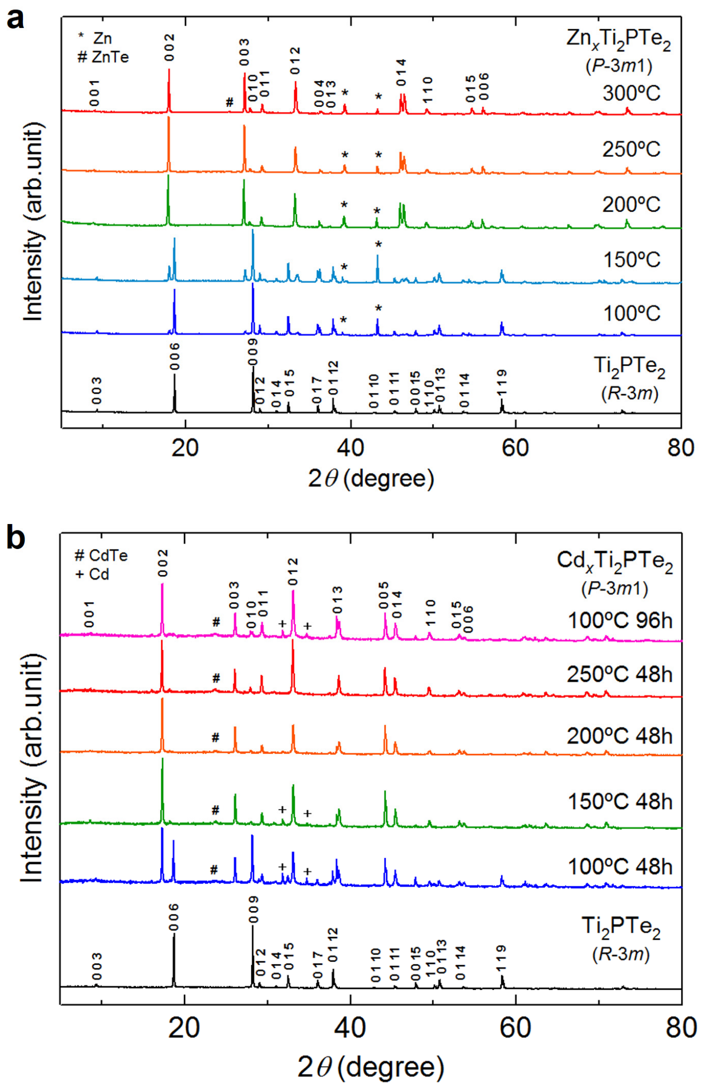

Supplementary Figure 12. Reaction temperature dependence of the intercalation reactions in Ti_2PTe_2. a, XRD patterns for the Zn-intercalated materials reacted at (from bottom to top) 100 °C, 150 °C, 200 °C, 250 °C, and 300 °C for 48 h. **b,** XRD patterns for the Cd-intercalated materials reacted at (from bottom to top) 100 °C, 150 °C, 200 °C, 250 °C for 48 h and 100 °C for 96 h. For both cases, with increasing reaction temperature, the amount of the metal-intercalated 1T phase increases relative to the 3R precursor phase.

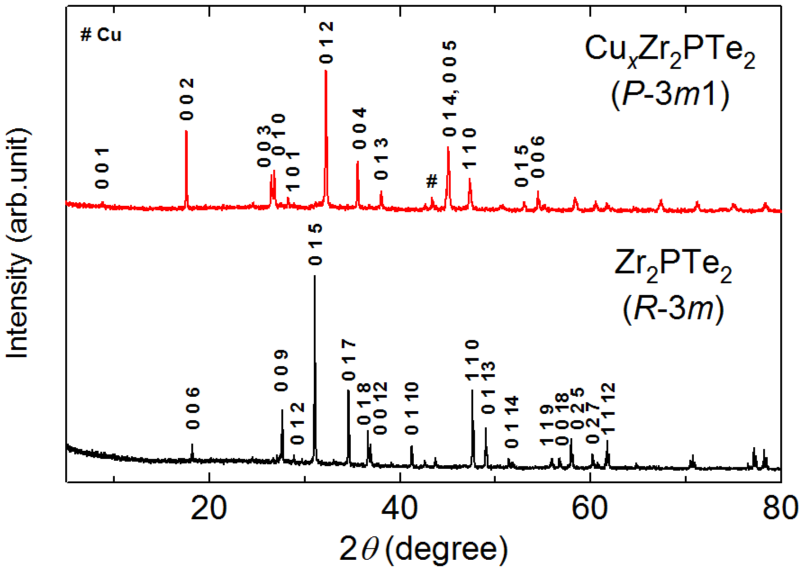

Supplementary Figure 13. XRD pattern for a Zr_2PTe_2 specimen reacted with Cu at 70 °C for 20 days. The lattice parameters of $Cu_xZr_2PTe_2$ are a = 3.8441(2) Å, c = 10.1076(4) Å.

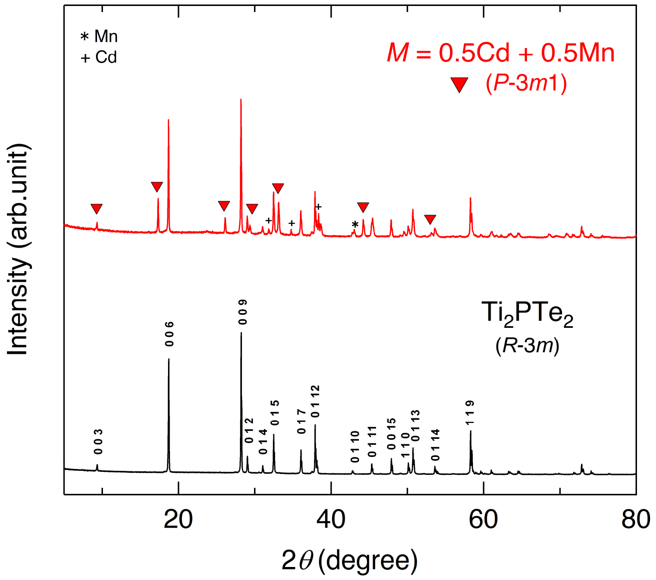

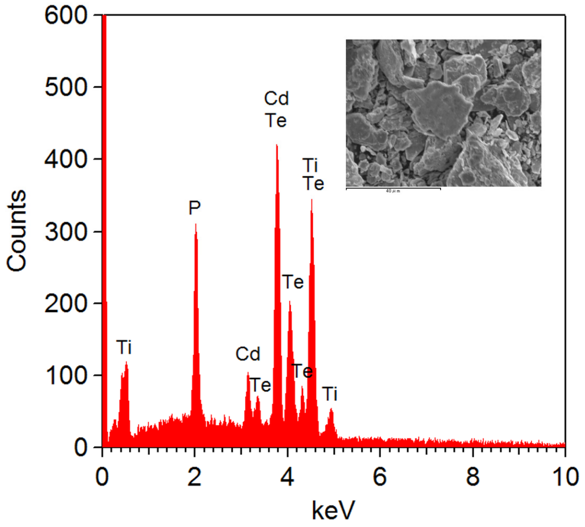

Supplementary Figure 14. XRD pattern and EDX spectra for a Ti_2PTe_2 specimen reacted with a mixture of Cd and Mn at 100 °C for 96 h. The molar ratio of Ti_2PTe_2 : Cd : Mn is 2 : 1 : 1. EDX spectra reveal Cd intercalation and the absence of Mn in the specimen, meaning selective removal (intercalation) of Cd. This spectrum gives the molar ratio Cd : Ti : P : Te = 0.26 : 2 : 1.1 : 1.8. The lattice parameters of $Cd_xTi_2PTe_2$ are a = 3.6779(2) Å, c = 10.2375(5) Å.

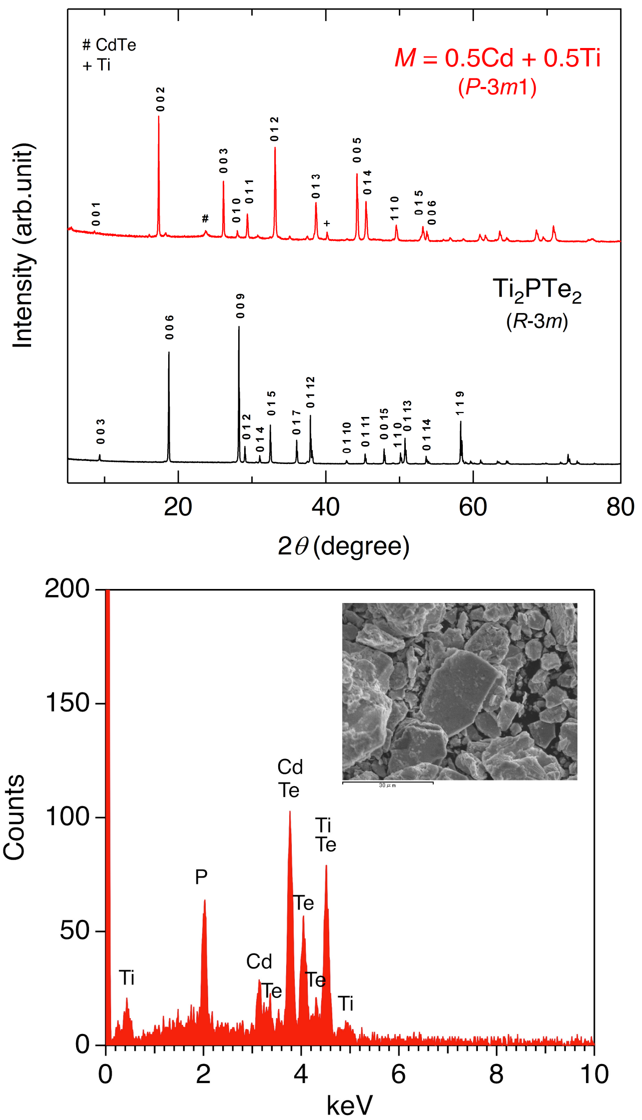

Supplementary Figure 15. XRD pattern and EDX spectra for a Ti_2PTe_2 specimen reacted with a powder mixture of Cd and Ti at 300 °C for 48 h. The molar ratio of Ti_2PTe_2 : Cd : Ti is 2 : 1 : 1. The EDX spectra revealed Cd intercalation and no extra Ti in the specimen, meaning selective removal (intercalation) of Cd. This spectrum gives the molar ratio Cd : Ti : P : Te = 0.30 : 2 : 1.0 : 2.2. The lattice parameters of $Cd_xTi_2PTe_2$ are a = 3.6789(1) Å, c = 10.2362(3) Å.

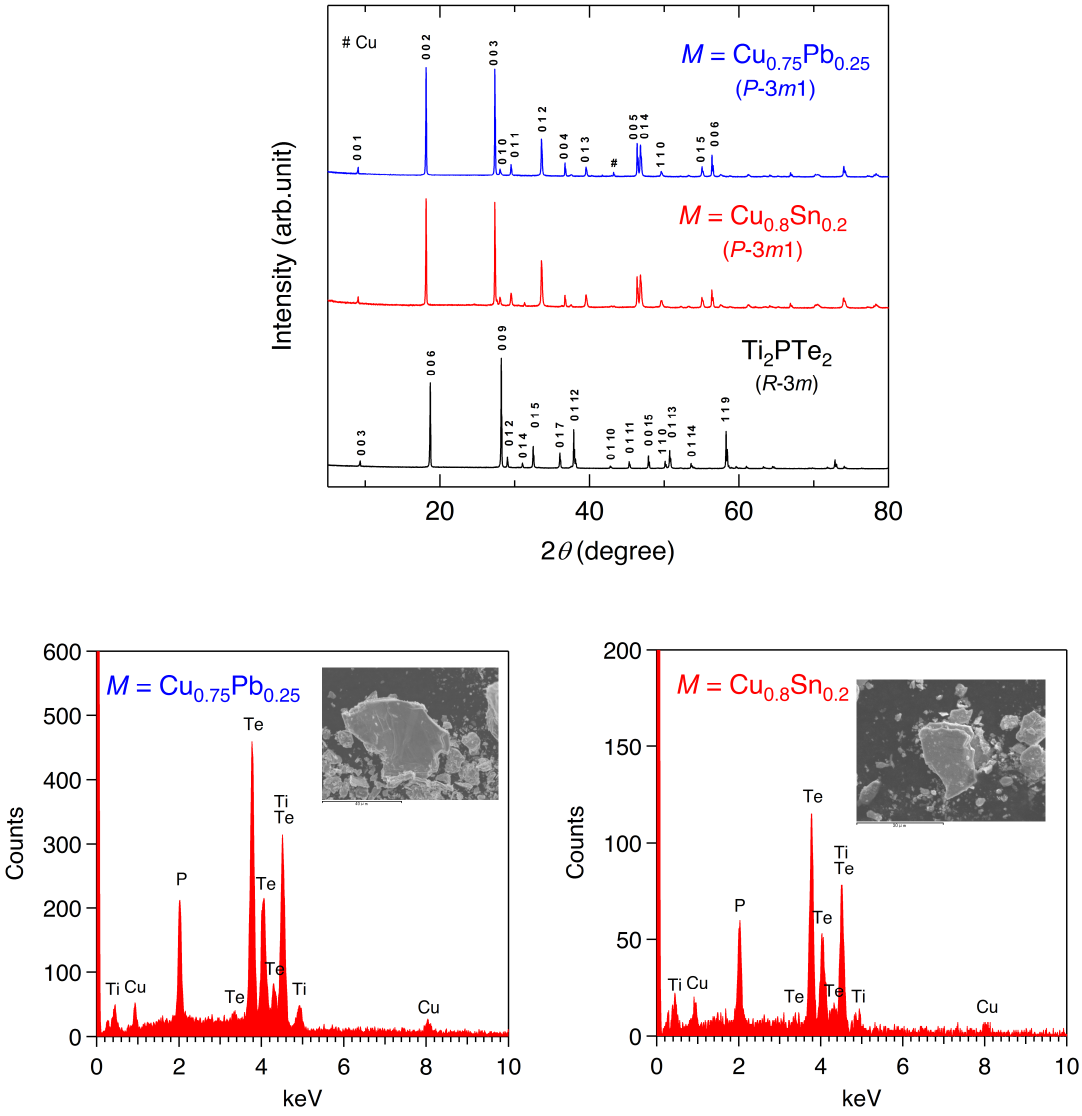

Supplementary Figure 16. XRD pattern and EDX spectra for a Ti_2PTe_2 specimen reacted with $Cu_{0.75}Pb_{0.25}$ alloy and with $Cu_{0.80}Sn_{0.20}$ alloy at 300 °C for 48 h. The molar ratio of Ti_2PTe_2: $Cu_{0.75}Pb_{0.25}$/$Cu_{0.80}Sn_{0.20}$ is 1 : 1. The EDX spectra revealed Cu intercalation and the absence of Pb and Sn in the specimen, meaning selective removal (intercalation) of Cu. Each spectrum gives the molar ratio Cu : Ti : P : Te = 0.33 : 2 : 0.91 : 2.1 and Cu : Ti : P : Te = 0.41 : 2 : 0.95 : 2.1. The lattice parameters of $Cu_xTi_2PTe_2$ are a = 3.67540(6) Å, c = 9.7857(2) Å ($Cu_{0.75}Pb_{0.25}$) and a = 3.67641(8) Å, c = 9.7891(2) Å ($Cu_{0.80}Sn_{0.20}$).

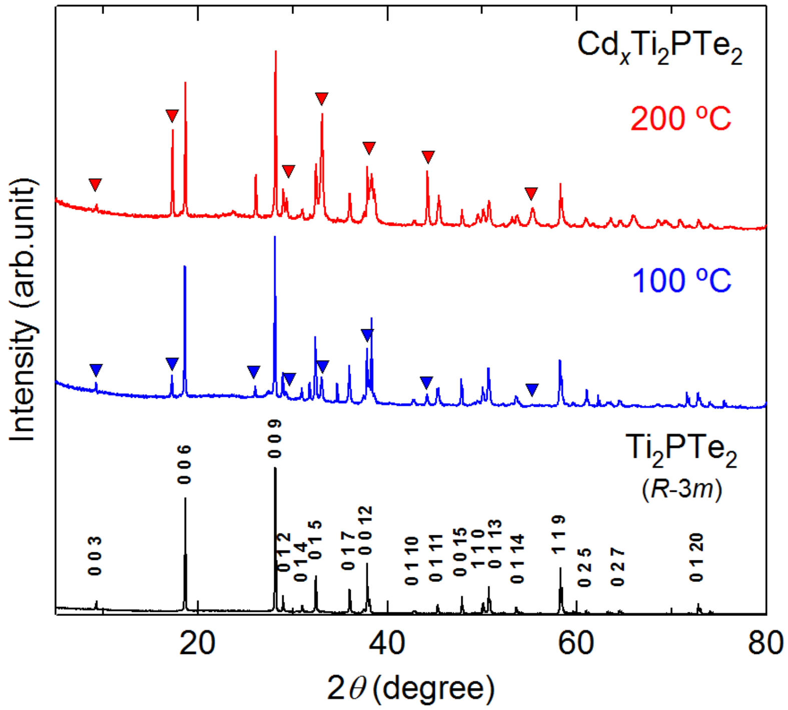

Supplementary Figure 17. Cd intercalation reactions with Ti_2PTe_2 performed in air. XRD patterns of Ti_2PTe_2 (bottom) and $Cd_xTi_2PTe_2$ obtained by the reaction with Cd powder at 100 °C (middle) and 200 °C (top) for 48 h in air. The triangles indicate the peaks of $Cd_xTi_2PTe_2$. The result is essentially the same as that obtained in vacuum.

Supplementary Table 1. Refined synchrotron X-ray (upper) and neutron (lower) structural parameters of $Zn_xTi_2PTe_2$.

Atom	Site	g	x	y	z	$100U_{eq}$ (Å^2)	$100U_{11}$ (Å^2)	$100U_{33}$ (Å^2)
Zn	2d	0.196 (3)<br>0.189(8)	1/3	2/3	0.489 (1)<br>0.461 (4)		0.028 (3)<br>0.10(2)	0.29 (2)<br>0.12 (2)
Ti	2d	1	1/3	2/3	0.1300 (1)<br>0.1286(4)	0.36(3)<br>0.85 (11)		
P	1a	1	0	0	0	0.49 (7)<br>0.13(11)		
Te	2d	1	1/3	2/3	0.68548 (8)<br>0.6856(3)	0.98 (2)<br>0.89(10)		

Space group P–3m1 (Z = 1). a = 3.68736(8) Å, c = 9.8380(2) Å, R_{wp} = 7.51%, R_p = 5.20% for SXRD refinement. a = 3.6858(3) Å, c = 9.8396(6) Å, R_{wp} = 6.36%, R_p = 4.95% for ND refinement. Anisotropic displacement parameters are used for the Zn site, where $U_{11} = U_{22} = ½U_{12}$ and $U_{13} = U_{23} = 0$.

Supplementary Table 2. Refined synchrotron X-ray structural parameters of $Cu_xTi_2PTe_2$ (upper), $Zn_xZr_2PTe_2$ (middle) and $Cd_xZr_2PTe_2$ (lower).

Atom	Site	*g*	*x*	*y*	*z*	100U_{eq} (Å^2)
Cu	2*d*	0.142(1)	1/3	2/3	0.4347 (4)	0.89 (26)
Ti	2*d*	1	1/3	2/3	0.13363 (9)	0.70 (2)
P	1*a*	1	0	0	0	0.92 (6)
Te	2*d*	1	1/3	2/3	0.68132 (5)	0.90 (1)

Atom	Site	*g*	*x*	*y*	*z*	100U_{eq} (Å^2)
Zn	2*d*	0.169 (3)	1/3	2/3	0.432 (1)	1.04 (30)
Zr	2*d*	1	1/3	2/3	0.1362 (1)	0.40 (4)
P	1*a*	1	0	0	0	0.83 (18)
Te	2*d*	1	1/3	2/3	0.6764 (1)	0.79 (4)

Atom	Site	*g*	*x*	*y*	*z*	100U_{eq} (Å^2)
Cd	2*d*	0.097(3)	1/3	2/3	0.505(2)	1.27
Zr	2*d*	1	1/3	2/3	0.1342(2)	0.63 (6)
P	1*a*	1	0	0	0	0.63
Te	2*d*	1	1/3	2/3	0.6823(2)	0.28 (5)

Space group *P*-3*m*1 (Z = 1). a = 3.67081(6) Å, c = 9.78199(9) Å, R_{wp} = 4.128%, R_p = 2.649% for $Cu_xTi_2PTe_2$, a = 3.8513(2) Å, c = 10.1528(4) Å, R_{wp} = 4.360%, R_p = 2.667% for $Zn_xZr_2PTe_2$, and a = 3.8429(3) Å, c = 10.4142(7) Å, R_{wp} = 5.374%, R_p = 2.850% for $Cd_xZr_2PTe_2$.

Supplementary Table 3. Refined parameters obtained from curve fitting of Zn K-edge EXAFS of $Zn_{0.4}Ti_2PTe_2$.

Anisotropic tetrahedral model based on the ND refined structure

Shell	*N*(Fixed)	*R* /Å	*DW* / Å
Zn-Te1	1	2.351	0.077
Zn-Te2	3	2.548	0.150

R_e: 2.407

Isotropic tetrahedral model

Shell	*N*(Fixed)	*R* / Å	*DW* / Å
Zn-Te1	4	2.331	0.126

R_e: 33.821

Octahedral model

Shell	*N*(Fixed)	*R* / Å	*DW* / Å
Zn-Te1	6	2.795	0.132

R_e: 56.089

The anisotropic tetrahedral model with two Zn-Te lengths (one short bond and three long bonds) is taken from the refined structural parameters by neutron diffraction. In the isotropic tetrahedral model, the position of Zn is set to give an equidistant Zn-Te bond. In the octahedral model, Zn is positioned at the 1*d* site (0, 0, 1/2). R_e represents the goodness of fit defined by:

$$R_e = \frac{\sum\left\{k^n\chi_{obs}(k) - k^n\chi_{cal}(k)\right\}^2}{\sum\left\{k^n\chi_{obs}(k)\right\}^2}, \quad \cdot\cdot\cdot (1)$$

where k, χ_{obs}, and χ_{cal} are the wave-vector, the experimental EXAFS signal and the calculated EXAFS signal, respectively.
